# High-throughput ovarian follicle counting by an innovative deep learning approach

**DOI:** 10.1038/s41598-018-31883-8

**Published:** 2018-09-10

**Authors:** Charlotte Sonigo, Stéphane Jankowski, Olivier Yoo, Olivier Trassard, Nicolas Bousquet, Michael Grynberg, Isabelle Beau, Nadine Binart

**Affiliations:** 1 Inserm U1185, Univ Parus Sud, Université Paris Sud, 94276 Le Kremlin Bicetre, France; 2 Quantmetry, 128 rue du Faubourg Saint Honoré, 75008 Paris, France; 3 INSERM, Institut Biomédical de Bicêtre, 80 rue du Général Leclerc, 94276 Le Kremlin Bicêtre, France; 4 Sorbonne Université, Laboratoire de Probabilité, Statistique et Modélisation, 4 place Jussieu, 75005 Paris, France; 5 Institut de Mathématiques de Toulouse, Université Paul Sabatier, 118 route de Narbonne, 31400 Toulouse, France; 6 Service de Médecine de la Reproduction et Préservation de la Fertilité, Hôpital Antoine Béclère, Hôpital Antoine Béclère, 92140 Clamart, France; 7 Univ Paris Sud, Université Paris-Saclay, 94276 Le Kremlin Bicetre, France

## Abstract

The evaluation of the number of mouse ovarian primordial follicles (PMF) can provide important information about ovarian function, regulation of folliculogenesis or the impact of chemotherapy on fertility. This counting, usually performed by specialized operators, is a tedious, time-consuming but indispensable procedure.The development and increasing use of deep machine learning algorithms promise to speed up and improve this process. Here, we present a new methodology of automatically detecting and counting PMF, using convolutional neural networks driven by labelled datasets and a sliding window algorithm to select test data. Trained from a database of 9 millions of images extracted from mouse ovaries, and tested over two ovaries (3 millions of images to classify and 2 000 follicles to detect), the algorithm processes the digitized histological slides of a completed ovary in less than one minute, dividing the usual processing time by a factor of about 30. It also outperforms the measurements made by a pathologist through optical detection. Its ability to correct label errors enables conducting an active learning process with the operator, improving the overall counting iteratively. These results could be suitable to adapt the methodology to the human ovarian follicles by transfer learning.

## Introduction

The ovary is endowed with a number of follicles established early in life. At any particular chronological age, the vast majority of oocytes in the ovary are present within non-growing primordial follicles (PMF). Reproductive aging is a continuous process involving the depletion that begins prior to birth and extends through the menopausal transition^1^. The PMF may actually have three different progressions: remaining quiescent for decades throughout the reproductive period; be activated and enter the growing process; or undergo atresia directly from the dormant stage^2^.

Each PMF is made of an oocyte arrested in meiotic prophase surrounded by a single layer of flat somatic cells, called granulosa cells. The process of follicle maturation is controlled by many growth factors and characterized by the combination of oocyte growth, proliferation of granulosa cells and formation of a fluid cavity, the antrum. From puberty, follicle can reach pre-ovulatory status and ovulates. In whole ovaries, follicles coexist at different development stages.

The activation or initial recruitment of PMF starts during fetal (in humans) or neonatal (in rodents) period and continues along reproductive life until the exhaustion of the follicular pool^3^. Despite recent advances, the precise mechanisms at play in the follicular activation remain ill-established. A balance between inhibitory and stimulatory factors may be crucial for maintaining the female reproductive lifespan^3^. Any disorder in the regulation process, due to genetic anomalies or gonadotoxic treatment for example, can lead to a premature loss of the follicular stockpile. The precise estimation of the remaining pool of non growing follicles represents a major issue. Indeed, such a tool could be critical to forecast the reproductive lifespan and predict future fertility. Furthermore, accurate models of reproductive aging would be relevant for women with a personal history of exposure to radiation, chemotherapy or prior ovarian surgery.

Yet, the actual number of PMF within ovaries can only be assessed using histological counting. The classical PMF counting, however, is a very complex, tedious, time consuming and operator dependent procedure^4^. Ovaries are serially sectioned and stained usually with hematoxylin-eosin and certain sections are manually analyzed by light microscopy for the presence of PMF. In order to improve this tedious procedure, corrective factors or evaluation of follicular density have been proposed. The complexicity of the procedure may explain the significant variations, up to 10 fold, in PMF count reported in the literature^4,5^.

The recent introduction of high‐resolution digital slide scanner system that acquires whole‐slide digital images offers the opportunity to quantify and improve histopathologic procedure with modern artificial intelligence methods^6^. Among these innovative technologies, deep learning is an actively emerging field regarding histological image analysis^7,8^. Deep learning techniques and more particularly Convolution Neural Networks (CNN) are state of the art techniques for image classification and pattern detection^9^. They are largely used nowadays for face recognition, image tagging, or even breast cancer diagnosis^10^. The increasing number of studies reporting deep learning based on image analysis demonstrates the growing interest of the pathologist community for this innovative technology. This method can accurately localize and classify cells into different cell types^11–13^. For example, deep learning approach holds great promise to improve breast and prostate cancer diagnosis and reduces the workload for pathologist^14,15^. Moreover, helping the diagnosis of HER2 status in breast cancer, this technique can provide useful informations to facilitate clinical decision making in affected patients^8^.

The present study explores, for the first time, deep learning approach for detecting and counting PMF in mouse ovaries. The objectives of this study were: (i) to develop and test a CNN-based methodology to automatically recognize and count PMF within mouse ovaries; (ii) to evaluate the performance of this procedure compared with a classical observer recognition and counting.

## Methods

### Animals

Twelve Swiss adult female mice were purchased by Janvier, France. Mice were group-housed under controlled conditions of temperature (21 °C ± 2 °C), and lighting (12 h light and 12 h dark cycles with lights-on at 0700 h), ad libitum access to food and water. Six-week-old animals were sacrificed by cervical dislocation and both ovaries were carefully removed. All procedures were approved by the local ethic committee Consortium des Animaleries Paris Sud (CAPSud) (N°2012–021).

### Tissue collection and processing

Ovaries were fixed in Bouin’s solution then embedded in paraffin blocks. Whole ovaries were serially cut into 4 µm sections using a microtome. One out of five sections was mounted on microscope slides and stained with Hematoxylin Eosin. Each slide contained around 7–8 ovarian sections.

### Manual follicle counting

**Figure 1:**
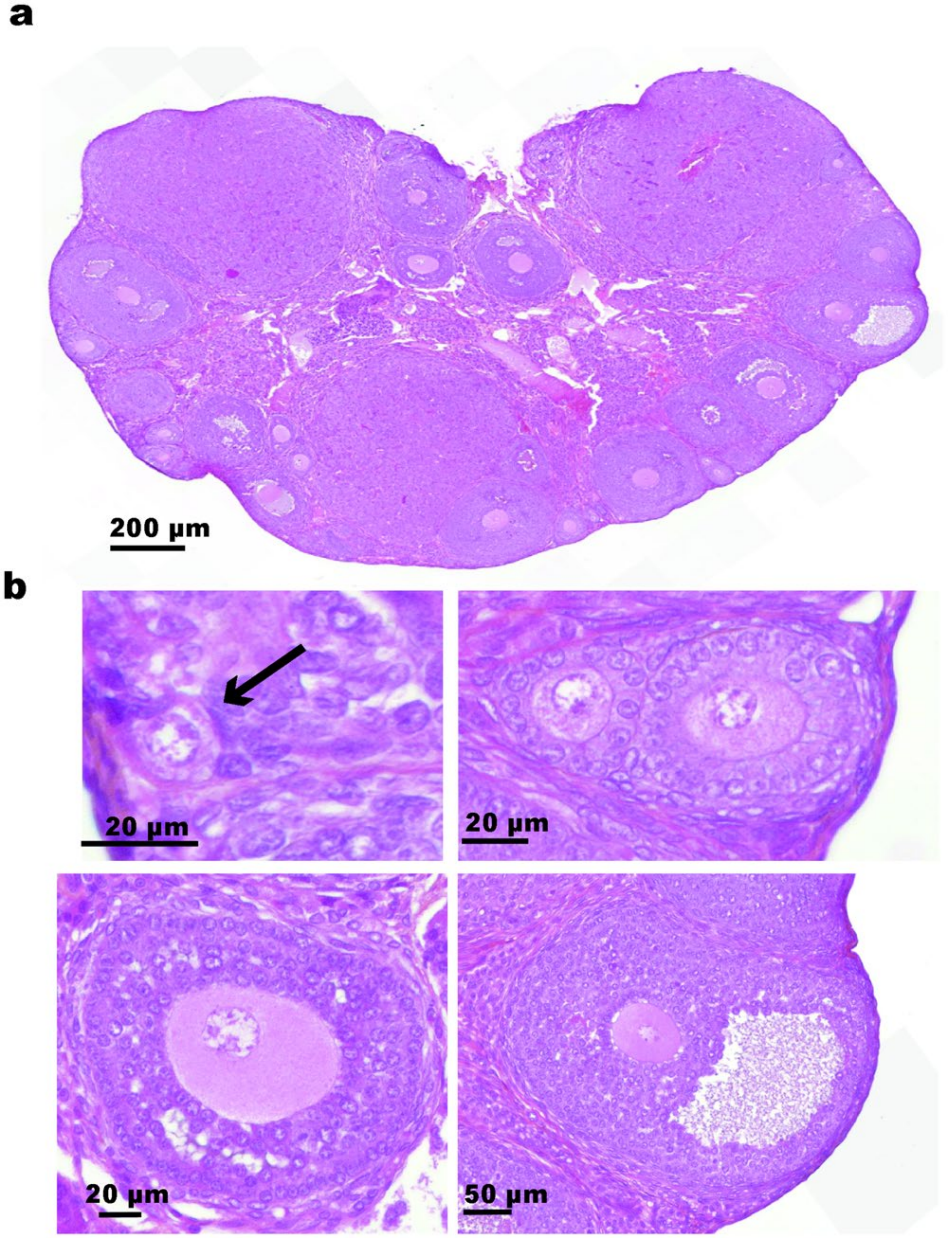
Identification of follicle type. (**a**) Example of one ovarian section stained with hematoxylin-eosin containing different classes of follicles. (**b**) Examples of a primordial follicle pointed by black arrow (left), constituted by an oocyte surrounding by some flattened granulosa cells; 2 primary follicles (right) constituted by an oocyte surrounded by a single layer of cuboidal granulosa cells. Below, a secondary follicle (left) with one oocyte surrounded by several layers of granulosa cells and an antral follicle (right) with a liquid cavity. Scale bars are shown.

The Panoramic 250 Flash, Slide Scanner (3DHISTECH Ltd. HUNGARY) system was used to capture digital whole slide high-resolution images (0.122 µm/pixel) with a 40x Plan-Apochromat objective and a 1.6x camera adapter magnification. Each section was analyzed using Calopix Viewer^®^, Follicles were classified according to Pedersen’s classification^16^. Briefly, follicles were classified as primordial if they contained an oocyte surrounded by a partial or complete layer of squamous granulosa cells; as primary follicles if they contained an oocyte surrounded by a single layer of cuboidal granulosa cells; as secondary follicles if at least 2 layers of granulosa cells were presented and as antral follicles if an antrum cavity was visualized (Fig. 1). To prevent double counting, a follicle was counted only when the nucleolus was identified. Using Calopix Viewer^®^, each follicle was annotated.

### Data extraction and preprocessing for automated follicle count

Using the software Pannoramic Viewer (3DHistech Ltd), the slides are extracted in a Tagged Image File Format (TIFF), suitable for analysis with usual deep learning tools. At the same time, the annotation coordinates from the manual follicle count were extracted with Calopix Viewer^®^. The result of this extraction is, for each slide, an Excel file containing every PMF, and its coordinates on the picture. This file is essential for the training of the supervised algorithm as it is used to automatically isolate the follicle images within the slide. Raw images extracted with the collection process presented above can approximate 200 GB in size, limiting the analytical possibilities. To reduce the resolution, thus the size of the input files, two following approaches were applied: (i) Resolution reduction: A compromise on image quality can be done to obtain lighter images that still allow the model to give good performances; (ii) Color removal: each pixel of a colored image is a combination of 3 values for red, green and blue, when black & white, each pixel is represented by one unique value, considerably reducing the size of the initial image. In addition, color does not seem to be an important factor for follicle detection, as long as contrast is preserved.

These size reduction techniques can be applied when extracting the TIFF images from Pannoramic Viewer. A LZW compression method was also used on the TIFF images to ease transfers and manipulations^17^.

### Deep learning tools

**Figure 2:**
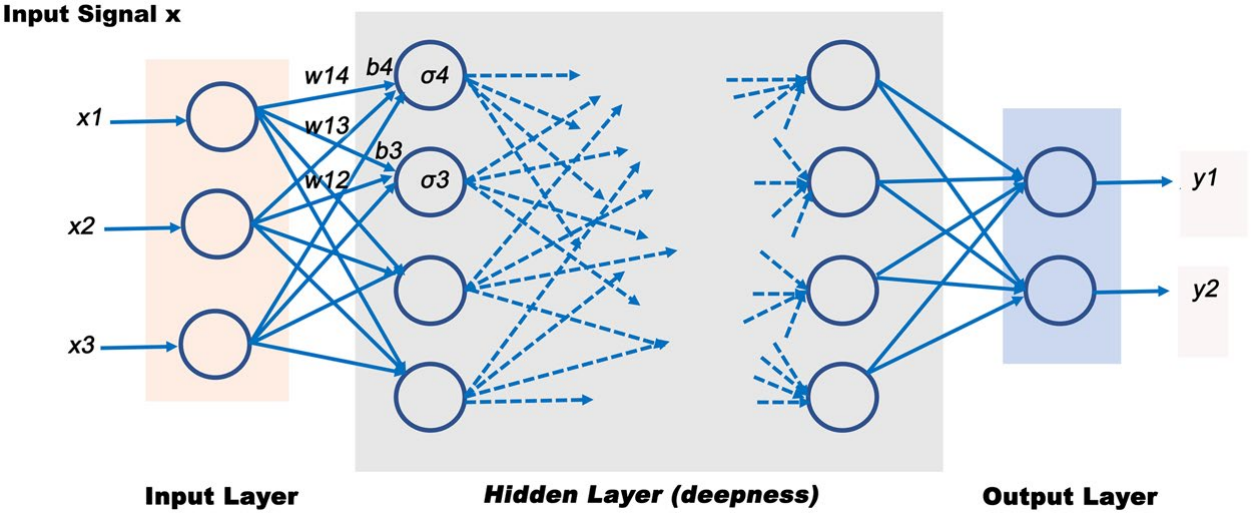
Illustration of an artificial neural network (ANN). A ANN is defined by successive layers, bias and activation functions, transforming a multivariate signal into a (possibly) multivariate output. *σ* is the activation function, bringing non linearity to the equation, *w*_*i*_ is the weight and *b* the bias

Deep learning is a specific branch of machine learning and involves deep neural networks which are artificial neural networks (ANN) with more than one hidden layer^18^. An ANN is composed of interconnected units disposed in successive layers, described as neurons (Fig. 2). In their most simple forms, these structures can learn information and approximate on a priori unknown mappings between a set of observed inputs and labelled outputs. Given an input signal (x_1_, …, x_n_), a neuron generates an output signal defined as

$$y=\sigma (\sum _{i=1}^{n}{w}_{i}{x}_{i}-b)$$

where *σ* is called the activation function, bringing non linearity to the equation, *w*_*i*_ is the weight and *b* the bias. Many functions can be used as activation functions, among them the sigmoid function and the Rectifed Linear Unit (ReLU) function, which is mostly used for deep architectures since it avoids the so-called vanishing gradient problem threatening the consistency of weight estimation by backpropagation (see below)^19^. Every layer of is composed of numerous neurons as described above.

More precisely, CNN were implemented, a type of feed-forward neural network known for its performance in computer vision tasks, as image analysis^18^. A CNN is usually composed of successive convolution and pooling layers, which act as features extractors, and generally ends with a fully-connected layer that aggregates this information. Each convolution layer can browse a fraction of an image and determine particular shapes by studying spatial dependencies between the different pixels of this fraction of the image, through the effects of a matrix of weights called a convolutional matrix or kernel. The output of a convolution layer is called a feature map. To reduce the dimension of a feature maps, pooling layers are usually used after convolutions. The classifier trained in this application is a custom implementation of a CNN mainly inspired by the VGG 19 architecture^20^, and the fully-connected layer that aggregates this information is produces the final output through the softmax activation function, defined in its *j-*th dimension by (for *j* = *1*, *…*, *d*)

$$\sigma {(z)}_{j}=\frac{{e}^{{z}_{j}}}{{\sum }_{k=1}^{d}{e}^{{z}_{k}}},$$

that provides a estimator of the vector probability of PMF presence in the sliding window.

Technical details about the architecture of the CNN, the nature of convolutional kernels and the pooling procedure are provided within the Supplementary Information (See Supplementary Figs S1–S3 and Supplementary Methods). The choice of this CNN laid on a preliminary study, where the classification power of two kinds of CNN (VGG versus the popular Alexnet architecture^21^) were compared over 1356 follicle images, with slightly better results for the VGG. Note besides that Region Based CNN^22^ and other Selective Search-based algorithms, as Faster RCNN^23^, which aim to locate an object within an image and to contour the neighboring area faster than using an iterative sliding window technique, could not be used here because they mostly require the complete image as an input; this problem could not be addressed our large medical images.

### Optimisation procedure

The optimisation of the CNN stands on the optimal assessment of weight (or convolutional) matrices from the training sample. The concept of backpropagation is key in this assessment and use of a neural network. During an iterative training phase, it is what makes the model more accurate every iteration. It uses the error made by the model at a given iteration to adjust the weights of the network. This progressive updating of weights to converge toward a more accurate model is represented by an optimization procedure. Different optimization techniques can be used to train a neural nets, mainly based on the stochastic gradient descent procedure.

For the optimization of our neural network a strategy of batch gradient descent was chosen, as it is a good trade-off between the traditional Stochastic Gradient Descent (SGD) and the batch gradient descent. Besides, the Adadelta optimization procedure was chosen^24^. Adadelta improves the gradient descent algorithm with the implementation of *momentum* and an adaptative learning rate. Moreover it does not require to initialize a learning rate, which makes it really convenient to use. To prevent overfitting in the assessment of the weights of the neural network, a dropout procedure was used^25^. Technical details about this procedure are provided within the Supplementary Fig. S4.

### Hardware and software specifications

All the code was created in Python 3. The following modules were used for specific treatments: (i) Keras to build the CNN^26^: It is a high-level module that runs on top of Tensorflow^27^, the deep learning library developed by Google Brain. (ii) OpenCV to manipulate images during the preprocessing phase^28^. It implements the contour detection algorithm and other specific functions for image manipulation. The code created for this project respects the best practices of Domain Driven Design^29^, aiming for a clear and well-structured code. The computer used to manipulate the images and to compute the CNN had a NVIDIA GeForce GTX 970 Graphical Processing Unit (GPU) with 1664 CUDA cores and 128 GB of RAM. The GPU allows better computing time for image processing and analysis. It is to be noted that more recent GPU could easily improve the computing performances of the project. The software specifications for this work allow the code to be easily reran on another computer or server since it only uses open source tools, developed for multiple operating systems. It does not depend on a specific hardware but the use of a last generation GPU and 128 GB of RAM is advised for good performances and reasonable execution time.

### Evaluation design and protocol

Two metrics were chosen to evaluate the performances of the model. The first one is the recall, representing the percentage of follicles actually found with the algorithm. This number is excpected to be as close as one as possible, meaning the algorithm does not miss any follicle. Note that considering the use of non maximum suppression, a frame containing two actual follicles will count as two found follicles (and not one true positive, as in precision). However, this recall metric needs to be balanced with a precision metric that takes into account the false positives of the model. Indeed a perfect recall can be obtained by predicting everything as a follicle, though not representing a good model. Precision is defined as the number of true positives (TP) divided by the sum of true positives and false positives (FP). TP is the number of frames predicted as follicles that actually contain a follicle and FP is the number of frames wrongly predicted as follicles. A precision close to 1 means that the algorithm tends to be right when it make a follicle prediction. Balancing the two metrics allow one to evaluate properly the overall quality of a model.

## Results

### Manual follicle counting

Twenty-four mouse ovaries were evaluated in this pilot study. A total of 194 slides were captured with the Slide Scanner and 1454 ovarian sections were analyzed by a single observer using Calopix Viewer^®^. All type of visualized follicles were classified according to Pedersen classification^16^ (Fig. 1) and specifically annoted using Calopix Viewer annotation system. Click annotation extraction in Excel format allows to count every follicles. A total of 40488 follicles were manually counted. Among them 21335 PMF were identified. Thus, each ovary contained an average of 888.9 ± 277.6 PMF. The time estimated for PMF counting was approximately one hour for one slide namely about 7–8 hours for one ovary.

### Data extraction

**Figure 3:**
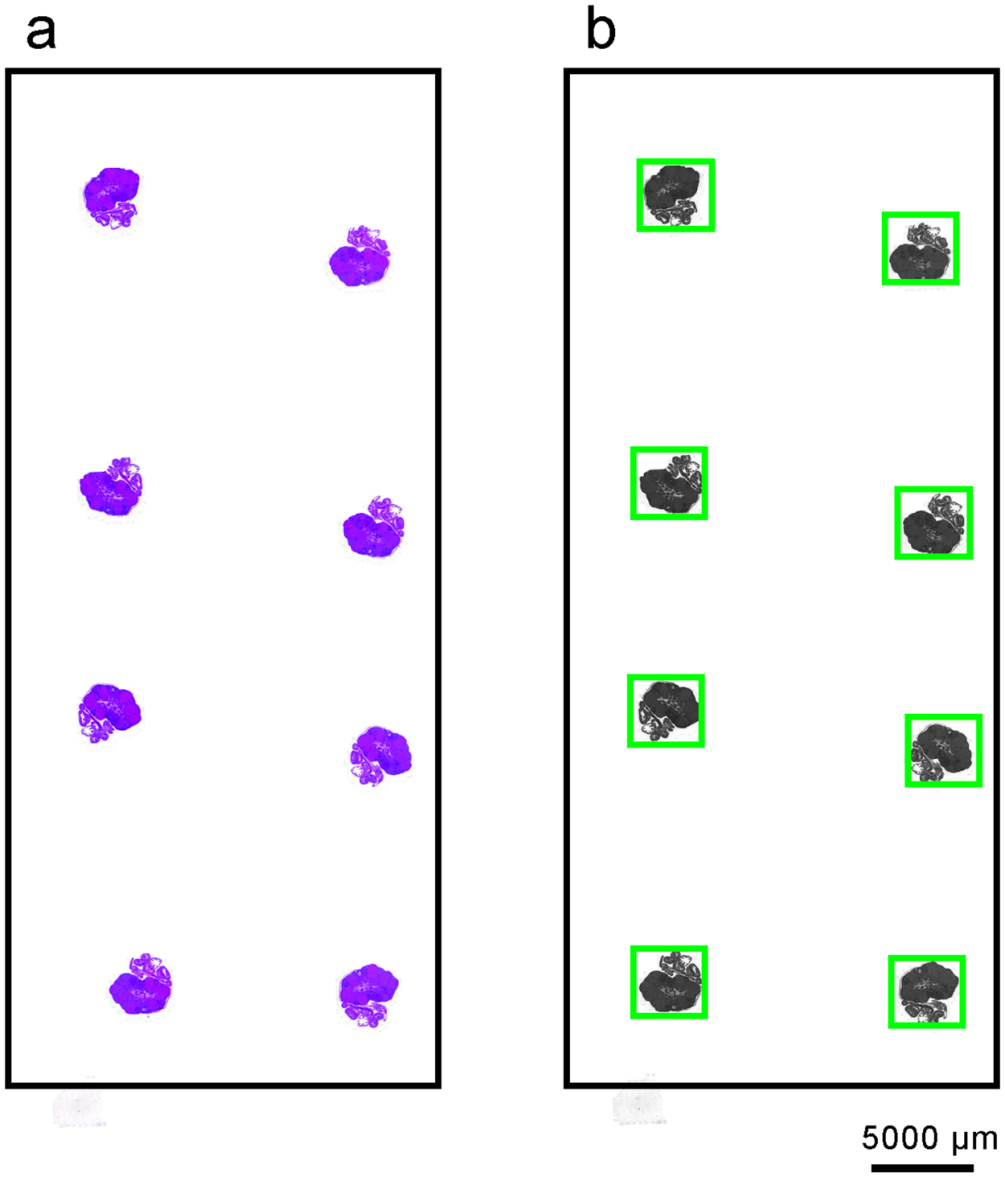
Scan of a slide containing ovarian sections. (**a**) Example of a raw slide containing 8 H&E stained sections from an ovary. (**b**) All sections are transformed individually as black and white images and isolated to remove the empty spaces.

Sixty-four slides containing 7–8 ovarian sections were extracted in TIFF image as represented in Fig. 3a. Therefore, the first step aimed to isolate every section into its own image in order to remove all the noise represented by the empty white spaces between the histological sections. For this purpose, the algorithm developed by Suzuki & Keiichi was used^30^. We applied the contour detection algorithm to a low resolution version of the slide in order to get better performance by preventing details and specificities of the sections to add noise to the detection process. The results of the detection were checked, both visually and by making sure no actual follicle was cropped out with this technique. An example of the application of this algorithm is reported on Fig. 3b. Finally, using the click coordinates from the manual counting, small frames containing the PMF can be extracted. They will represent the positive examples in the supervised methodology described below. The size of the frame is constant for all PMF and empirically calculated to make sure it always contains the PMF. Many negative examples, with the same frame size, were randomly picked in the cut images to balance the dataset.

A last phase of data augmentation was conducted to increase the relevance of the dataset for feeding a detection and classification algorithm. It stands on the fact that some image distortions produces other relevant images for the study (namely, images that could have been observed too)^31^. In our context only the following geometrical distorsions were applied to increase the dataset: rotations, image translation and horizontal and vertical image reversals.

### Developing and training a deep learning approach

**Figure 4:**
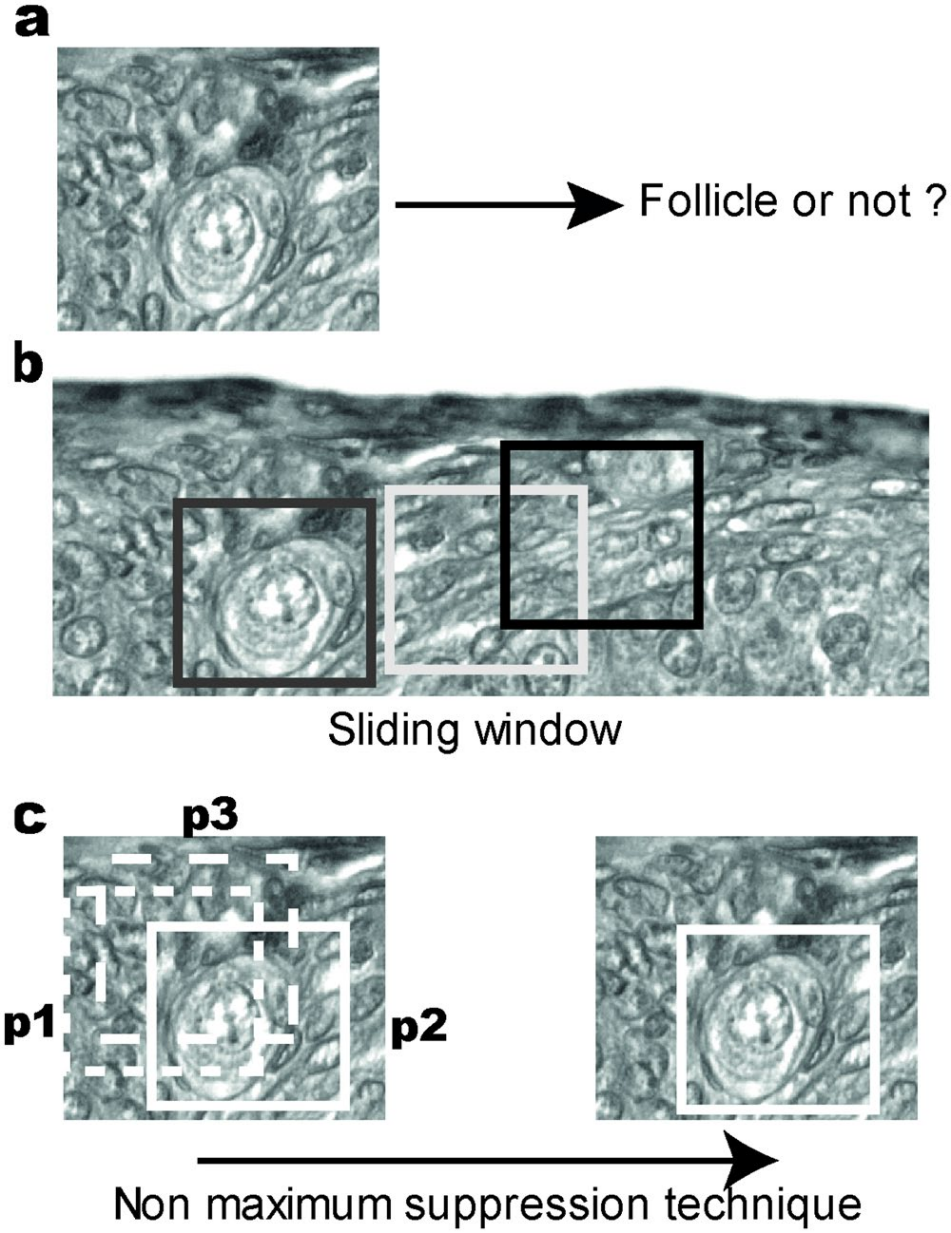
Deep learning training steps. (**a**) First step is a training of follicle detection. (**b**) In the second step, sliding windows (grey, white and black squares) scan the cut image and use the previous step to detect or not detect follicles. (**c**) Illustration of overlapping and application of the non maximum suppression technique. The windows (white dotted lines) with adjacent frames are computed in a probability vector (p_1_, p_2_, p_3_) of PMF presence by the neural network, with p_1_ < p_2_ and p_3_ < p_2_. The imputation results of windows 1 and 3 are then removed from the detection process. This process aims to keep only the frame giving the maximum probability of follicle.

In the present study, 48 slides corresponding to 6 whole ovaries were used to develop and train the algoritm process. Follicle detection was defined as a two-stages process. First, a CNN was trained to classify whether an image contains a follicle or not, as illustrated in Fig. 4a. The training dataset contains small frames of both real follicles and random parts of the ovary section. Secondly, a sliding window was used to analyze the image and, for each image extracted by this way, predicted the presence of a follicle using the model previously trained. The window was the same size as the frames previously used to train the CNN (Fig. 4b). Note that, in the prediction process, successive windows overlap by half to make sure that no follicle is missed due to position in the window (for example section between two windows or in an edge of the window). However this overlap can generate multiple positive frames for the same follicle. This could lead to an overestimation of the performance metrics with artificial true positive examples. To avoid this problem, a non maximum suppression technique^32^ was implemented. It aims to keep only the frame giving the maximum probability of follicle within multiple adjacent positive frames, thus avoiding the redundancy problem (see Fig. 4c for an illustration).

### Reliable follicle counts with the automated procedure

Two whole ovaries were used as a testing set, resulting in 2 875 160 images from the 15 slides available. A total of 1667 PMF were manually counted and annotated. The training time to process one ovary is around 1 hour, with small variations depending on the number of slides and sections per slide (typically 1 slide is processed in 7 minutes on average). Note that every True Positives (TP) and False Positives (FP) found during the prediction was saved as well as a more global cut image with squares surrounding the found follicles. This saving aspect had a significant contribution to the overall prediction time of the algorithm.

The model outputed, for one frame, a probability of containing a PMF. A threshold must be set to discriminate between positives and negatives. Intuitivelly, variations of this threshold will influence the number of TP and FP and so recall and precision.

Remind that recall, or sensitivity, is defined in the present context by

$$recall=\frac{number\,of\,correctly\,detected\,follicles\,}{number\,of\,real\,follicles},$$

while the precision is defined by

$$precision=\frac{number\,of\,correctly\,detected\,follicles\,}{number\,of\,detected\,follicles}.$$

For this study, a relatively low threshold was chosen in order to keep a high recall, thus finding nearly all the actual PMF, but at the cost of many FP. A recall of 99.46% was found with a precision of 11.32%. This means that 1658 out of 1667 follicles were found whereas only one out of ten positive prediction from the CNN was a right one (nearly 89% of detected follicules were FP).

A hard negative mining (HNM) methodology^33^ was implemented in order to reduce the huge number of false positive. It consists in, after a first phase of training, adding false positive from a specific HNM dataset forced as negative examples in the training set and continuing with training. The negative examples of the training set are resampled to avoid training on unbalanced data. HNM encourages the model to focus on examples that still trick the model after a first step of training and so to avoid FP in the future. This procedure considerably improves the precision metric. However, it also has a negative impact on the recall and needs to be used carefully. After the HNM implementation, precision was 57.38% for a recall of 90.40% (nearly 43% of detected follicules were FP). Finally, note that some of the FP remaining in the final prediction of the algorithm are actual follicles missed during the fastidious manual counting. After a manual check on the FP generated by the model, 185 turned out to be missed follicles, that being 19,5% of all the FP. Considering this, the final results were corrected, giving a recall of 91,36% with a precision of 65,69%. All the results at the different steps of the evaluation process can be viewed in Table 1.

**Table 1:** Different steps of the evaluation process.

	Phase I (after training)	Phase II (after training and HNM)	Phase III (after training, HNM and operator focus)
Total number of images	2 875 160	2 875 160	2 875 160
Number of false positive	14 053	949	949–185
% of false positive	0.49%	0.044%	0.037%
Number of true negative	2 859 304	2 872 421	2 872 421
% of true negative	99.45%	99.90%	99.90%
Precision (%)	11.32%	57.38%	65.69%
Recall (%)	99.46%	90.40%	91.36%
**Number of detected follicles**	**1 658**	**1 507**	**1692** **(1 507** + **185)**

Data were based on 1667 manually counted follicles.

HNM: Hard Negative Mining.

## Discussion

In the present study, histological analysis was improved through the development of whole slide scanning system that digitizes slides with stained tissue sections at high resolution. Further, whole‐slide digital images, obtained through this process, offers the application of innovative image analysis techniques for the histopathological examination. Among these techniques, deep learning is a modern artificial intelligence method which emerged in the field of histopathology over the past few years. This CNN allows automated quantification of various parameters on slides^8,11,34^. In the present study, we applied for the first time these two techniques to evaluate the follicular content and especially the PMF count in mouse ovaries. We showed that deep learning approach allowed PMF recognition and subsequently counting within serially ovarian sections.

Classically, slides are analyzed with microscope and follicle count is generated manually on multiregistered cell counter^35^. Subsequently, the data is recovered and transferred to a computer database. This technique is laborious, costly, time consuming and highly operator-dependent. As a result, over the past decades, efforts were made to improve this procedure and different ovarian follicle counting protocols were investigated. At first, authors proposed to count follicles not in all sections but only in every fifth to tenth sections. For example, a study compared the efficiencies of various sampling paradigms to define sensitive and less tedious means to obtain differential follicle counts and concluded that follicle counts from 1% to 5% random samples may provide a good estimation of ovarian toxicity in mice^35^. Accepting that this sampling methodology provides a reliable estimate of the number of follicles number per ovary, researchers proposed to apply a corrective factor to account for the proportion of the ovary not included in the sample. This corrective factor varies in literature from 5 to 80, each time with “rational” explanation^4^. Nonetheless, the number of follicles varied by 10-fold or more depending on the study and this technique was not reproducible. Other methods, such as the use of fractionator/physical dissector technique have been evaluated to determine the number of follicles in mice^5^, non-human primate^36^ or human ovaries^37^. This technique based on stereology techniques avoids the introduction of corrective factors and seems more efficient and less time-consuming^37^. Nevertheless, this technique needs the use of an oil immersion objective on microscope and the follicle counting still remains manual and operator dependent.

In the present study, we proposed, for the first time, an automatized technique for PMF counting using deep learning approach. We analyzed every fifth ovarian section, even this sectioning was not always in equatorial plane, very few PMFs might be forgotten. This method is efficient and accurate to evaluate the PMF number. Moreover, it outperforms manual procedures since it is time saving and allows the detection of some PMFs overlooked by the operator. In addition, this algorithm makes it possible to highlight any errors in the operator’s classification. The latter can then act in turn as an oracle, checking certain false positive predictions. This iterative process of active learning between the algorithm and the operator allows for increase reliability of counting and learning.

Several avenues of research, which will be the subject of future work, are likely to further increase the operational scope of this study. First, the implementation of the exploration and classification algorithm currently requires the conversion of images originally created in MRXS to TIFF. This operation is currently very expensive in terms of computation time but will benefit from the forthcoming arrival of new technological improvements.

Above all, the binary classification operated by the algorithm could be extended. For this pilot study, we choose to count only PMFs as these follicles are the most abundant follicular population and are difficult to detect in ovarian cortex. Nevertheless, they reflect the ovarian reserve that is crucial to assess the fertility in pathological conditions. However, this counting of only one class of follicles represents a limitation of the present study as several follicular classes (primary, secondary and antral follicles including atretic follicles) are present within ovaries. Counting all follicles within ovaries and differentiating each follicle type is an important issue for some fertility studies and the count of all follicular classes is needed and a new procedure should be developed. This could be carried out by using a softmax activation function. It would require more and finer labelled data, but also a slight increase in the number of convolution and pooling layers. This would make it possible to differentiate follicles according to a greater number of patterns hidden in the images. The reduction of processing time mentioned above is therefore an important factor for the success of this approach. Moreover, another option might be to develop a 3D method and construct a specific algorithm based on the same deep learning approach, to count all follicles classes.

The development of an accurate method to count PMF into human ovaries represents a great challenge to investigate, for example, aging process. The implementation of this methodology for counting human ovarian follicles could feed on the close proximity between human follicles and mouse follicles. If human follicles are larger than mouse follicles, their morphology remains similar. It would therefore seem appropriate to use a CNN calibrated on mouse data to pre-train a CNN dedicated to human data processing (transfer learning process), treating hyperparameters related to size and shape of objects differently. In particular, an area of interest for human might be PMFs count in ovaries from patients facing with cancer. Indeed, ovarian cortex cryopreservation is a new effective technique of fertility preservation for both prepubertal girls and women in reproductive age^38^. The subsequent graft of cortex fragments allows the restoration of ovarian function and possible natural pregnancies^39^. The precise evaluation of the number of PMF within these ovaries might allow to better inform patients and improve the effectiveness of the technique.

## Electronic supplementary material

Supplementary Information

## References

[1] Wallace WHB, Kelsey TW (2010). Human ovarian reserve from conception to the menopause. PloS One.

[2] Reddy P, Zheng W, Liu K (2010). Mechanisms maintaining the dormancy and survival of mammalian primordial follicles. Trends Endocrinol. Metab. TEM.

[3] Monniaux D (2014). The ovarian reserve of primordial follicles and the dynamic reserve of antral growing follicles: what is the link?. Biol. Reprod..

[4] Tilly JL (2003). Ovarian follicle counts–not as simple as 1, 2, 3. Reprod. Biol. Endocrinol. RBE.

[5] Myers M, Britt KL, Wreford NGM, Ebling FJP, Kerr JB (2004). Methods for quantifying follicular numbers within the mouse ovary. Reprod. Camb. Engl..

[6] Casari C (2013). Accelerated uptake of VWF/platelet complexes in macrophages contributes to VWD type 2B-associated thrombocytopenia. Blood.

[7] Sharma, H., Zerbe, N., Klempert, I., Hellwich, O. & Hufnagl, P. Deep convolutional neural networks for automatic classification of gastric carcinoma using whole slide images in digital histopathology. *Comput. Med. Imaging Graph. Off. J. Comput. Med. Imaging Soc*. 10.1016/j.compmedimag.2017.06.001 (2017). 10.1016/j.compmedimag.2017.06.001 28676295

[8] Vandenberghe ME (2017). Relevance of deep learning to facilitate the diagnosis of HER2 status in breast cancer. Sci. Rep..

[9] LeCun Y, Bengio Y, Hinton G (2015). Deep learning. Nature.

[10] Araújo T (2017). Classification of breast cancer histology images using Convolutional Neural Networks. PloS One.

[11] Cireşan DC, Giusti A, Gambardella LM, Schmidhuber J (2013). Mitosis detection in breast cancer histology images with deep neural networks. Med. Image Comput. Comput.-Assist. Interv. MICCAI Int. Conf. Med. Image Comput. Comput.-Assist. Interv..

[12] Su H (2015). Robust Cell Detection and Segmentation in Histopathological Images Using Sparse Reconstruction and Stacked Denoising Autoencoders. Med. Image Comput. Comput.-Assist. Interv. MICCAI Int. Conf. Med. Image Comput. Comput.-Assist. Interv..

[13] Sirinukunwattana K (2016). Locality Sensitive Deep Learning for Detection and Classification of Nuclei in Routine Colon Cancer Histology Images. IEEE Trans. Med. Imaging.

[14] Litjens G (2016). Deep learning as a tool for increased accuracy and efficiency of histopathological diagnosis. Sci. Rep..

[15] Bejnordi BE (2017). Diagnostic Assessment of Deep Learning Algorithms for Detection of Lymph Node Metastases in Women With Breast Cancer. JAMA.

[16] Pedersen T, Peters H (1968). Proposal for a classification of oocytes and follicles in the mouse ovary. J. Reprod. Fertil..

[17] Welch TA (1984). A Technique for High-Performance Data Compression. Computer.

[18] Goodfellow, I., Bengio, Y. & Courville, A. Deep Learning (Adaptive Computation and Machine Learning series), part II, chapter 6, (The MIT press 2016).

[19] Hochreiter, S., Bengio, Y., Frasconi, P. & Schmidhuber, J. A field guide to dynamical recurrent networds, Wiley-IEEE Press, chapter: *Gradient Flow in Recurrent Nets: the Difficulty of Learning Long-Term Dependencies* (2001).

[20] Simonyan, K. & Zisserman, A. Very Deep Convolutional Networks for Large-Scale Image Recognition. *ArXiv14091556 Cs* (2014).

[21] Krizhevsky, A., Sutskever, I. & Hinton, G. E. ImageNet Classification with Deep Convolutional Neural Networks. In *Advances in Neural Information Processing Systems* 25 (eds Pereira, F., Burges, C. J. C., Bottou, L. & Weinberger, K. Q.) 1097–1105 (Curran Associates, Inc., 2012).

[22] Girshick, R., Donahue, J., Darrell, T. & Malik, J. Rich Feature Hierarchies for Accurate Object Detection and Semantic Segmentation. in *Proceedings of the 2014 IEEE Conference on Computer Vision and Pattern Recognition* 580–587, 10.1109/CVPR.2014.81 (IEEE Computer Society2014).

[23] Ren, S., He, K., Girshick, R. & Sun, J. Faster R-CNN: Towards Real-Time Object Detection with Region Proposal Networks. *ArXiv150601497 Cs* (2015). 10.1109/TPAMI.2016.2577031 27295650

[24] Zeiler, M. D. ADADELTA: An Adaptive Learning Rate Method. *ArXiv12125701 Cs* (2012).

[25] Srivastava N, Hinton G, Krizhevsky A, Sutskever I, Salakhutdinov R (2014). Dropout: A Simple Way to Prevent Neural Networks from Overfitting. J. Mach. Learn. Res..

[26] Chollet, F. Keras, GitHub. https://github.com/fchollet/keras (2015).

[27] Abadi, M. *et al*. TensorFlow: Large-Scale Machine Learning on Heterogeneous Distributed Systems. *ArXiv160304467 Cs* (2016).

[28] Bradski, G. The OpenCV Library. Dr. Dobb’s Available at: http://www.drdobbs.com/open-source/the-opencv-library/184404319. (Accessed: 16th February 2018).

[29] Evans, E. *Domain-Driven Design: Tackling Complexity in the Heart of Software*. (2013).

[30] Suzuki S, be K (1985). Topological structural analysis of digitized binary images by border following. Comput. Vis. Graph. Image Process..

[31] Perez, L. & Wang, J. The Effectiveness of Data Augmentation in Image Classification using Deep Learning. *ArXiv171204621 Cs* (2017).

[32] Felzenszwalb PF, Girshick RB, McAllester D, Ramanan D (2010). Object Detection with Discriminatively Trained Part-Based Models. IEEE Trans. Pattern Anal. Mach. Intell..

[33] Dalal, N. & Triggs, B. Histograms of oriented gradients for human detection. In *2005 IEEE Computer Society Conference on Computer Vision and Pattern Recognition* (CVPR’05) **1**, 886–893 (2005).

[34] Wang, H. *et al*. Mitosis detection in breast cancer pathology images by combining handcrafted and convolutional neural network features. *J. Med. Imaging Bellingham Wash* **1**(3) (2014) 10.1117/1.JMI.1.3.034003 PMC4479031 26158062

[35] Bucci TJ, Bolon B, Warbritton AR, Chen JJ, Heindel JJ (1997). Influence of sampling on the reproducibility of ovarian follicle counts in mouse toxicity studies. Reprod. Toxicol. Elmsford N.

[36] Miller PB, Charleston JS, Battaglia DE, Klein NA, Soules MR (1997). An accurate, simple method for unbiased determination of primordial follicle number in the primate ovary. Biol. Reprod..

[37] Charleston JS (2007). Estimating human ovarian non-growing follicle number: the application of modern stereology techniques to an old problem. Hum. Reprod. Oxf. Engl..

[38] Guzy, L. & Demeestere, I. Assessment of ovarian reserve and fertility preservation strategies in children treated for cancer. *Minerva Ginecol*. (2016). 10.23736/S0026-4784.16.03992-7 27787477

[39] Donnez J, Dolmans M-M (2017). Fertility Preservation in Women. N. Engl. J. Med..

