# Supplementary material for: High-throughput ovarian follicle counting by an innovative deep learning approach
Source: Sci Rep. 2018 Sep 10;8:13499. doi: 10.1038/s41598-018-31883-8 (PMC6131397; doi:10.1038/s41598-018-31883-8)
Supplement: Supplementary file 1 — Supplementary Information [file 41598_2018_31883_MOESM1_ESM.docx]

**High-throughput ovarian follicle counting by an innovative deep learning approach**

Charlotte Sonigo^1^, Stéphane Jankowski^2^, Olivier Yoo^2^, Olivier Trassard^3^, Nicolas Bousquet^2,4,5^, Michael Grynberg ^6,7^, Isabelle Beau^1^ and Nadine Binart^1^

^1^ Inserm U1185, Faculté de médecine Paris-Sud, Université Paris Saclay, Le Kremlin Bicêtre, 94276, France

^2^ Quantmetry, 128 rue du Faubourg Saint Honoré, 75008 Paris, France

^3^ INSERM, Institut Biomédical de Bicêtre, 80 rue du Général Leclerc, Le Kremlin Bicêtre, 94276, France

^4^ Sorbonne Université, Laboratoire de Probabilité, Statistique et Modélisation, 4 place Jussieu, 75005 Paris, France

^5^ Institut de Mathématique de Toulouse, Université Paul Sabatier, 118 route de Narbonne, 31400 Toulouse, France

^6^ Department of Reproductive Medicine and Fertility Preservation, Hôpital Antoine Béclère, 92140 Clamart, Université Paris Saclay, Le Kremlin Bicêtre, 94276, France

^7^ University Paris Sud XI, 94276, Le Kremlin Bicêtre, France

Correspondance to : Dr Charlotte Sonigo, Inserm U1185, Université Paris Saclay, Le Kremlin Bicêtre, 94276, France. Phone : +33149596702 ; Fax: +33149596732 ; Email: charlotte.sonigo@gmail.com

**Supplementary Method. Max-pooling.** Pooling layers are frequently used after convolution layers to reduce feature maps and avoid over-learning by synthesizing the information transmitted by these maps while significantly reducing the overall computational cost. Indeed they do not require any weight training. A pooling layer splits the image (or a fraction of an image in the case of CNN) into a series of non-overlapping rectangles of n x p pixels, each of them being affected a global value defined as the output of a function of the values of all pixels belonging to the rectangle. In this work, an usual choice of n x p = 2 x 2 rectangles procedure was made to minimize the loss of information by choosing the common max-pooling procedure (selecting the highest pixel value in the rectangle), known to provide robustness to local deviations of features and switching between activation subsets.

**
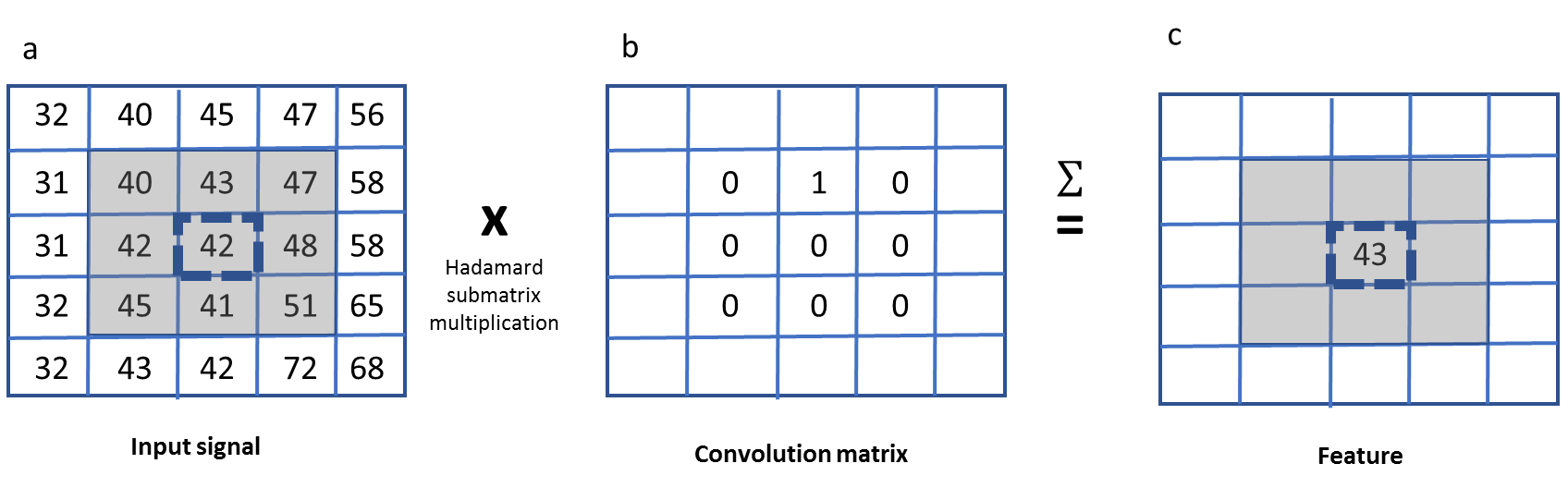
**

**Supplemental Figure S1. Simple Convolutional kernels.** In the discretized world of image processing, a convolutional kernel is a matrix of weights transforming a fraction of an input image, described as an array of pixels, into a feature of ordinarily smaller dimension (the ensemble of feature being called a *feature map*). The matrix of weights is designed in order to favor features exhibiting particular spatial dependencies between pixels, the complexity of which increasing with the depth of the convolutional layer. The left matrix (**a**) describes an image where a group of pixels (in grey) is selected. The convolutional network browses successively each pixel of the submatrix (called “initial” pixel) and multiply its value and the value of the 8 neighboring pixels by the corresponding value within the kernel (**b**), then add the results. In the example, the initial pixel in left is delimited by dashed lines. The graphical result of the submatrix multiplication is a one-pixel downward shift of the initial pixel (**c**).


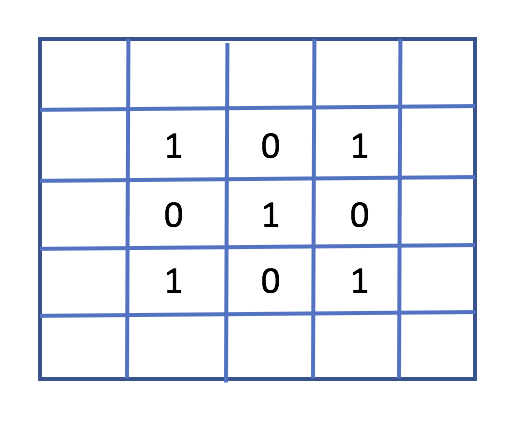


**Supplemental Figure S2. A more elaborated convolution kernel.** Here, the kernel is designed to detect cross-shaped patterns within the pixels. This type of more elaborated kernel, for instance, can be structured to detect cross-shaped patterns.

In the present work, as usually for neural network techniques, the kernels were automatically learned within the training phase, by comparing predicted and observed labels and using backpropagation. More generally, a CNN is determined by the dimensions (W,H) of the kernel (width and height of the convolution matrix), its depth D (the number of feature maps), its stride S (measuring the overlapping of kernels browsing the image ; the largest, the weakest overlapping) and the padding P, which mainly controls the dimension of images, maps and output. It can be used to generate artificial pixels around the input image (or fraction of image) such that the dimensions of the image and the feature map be similar.


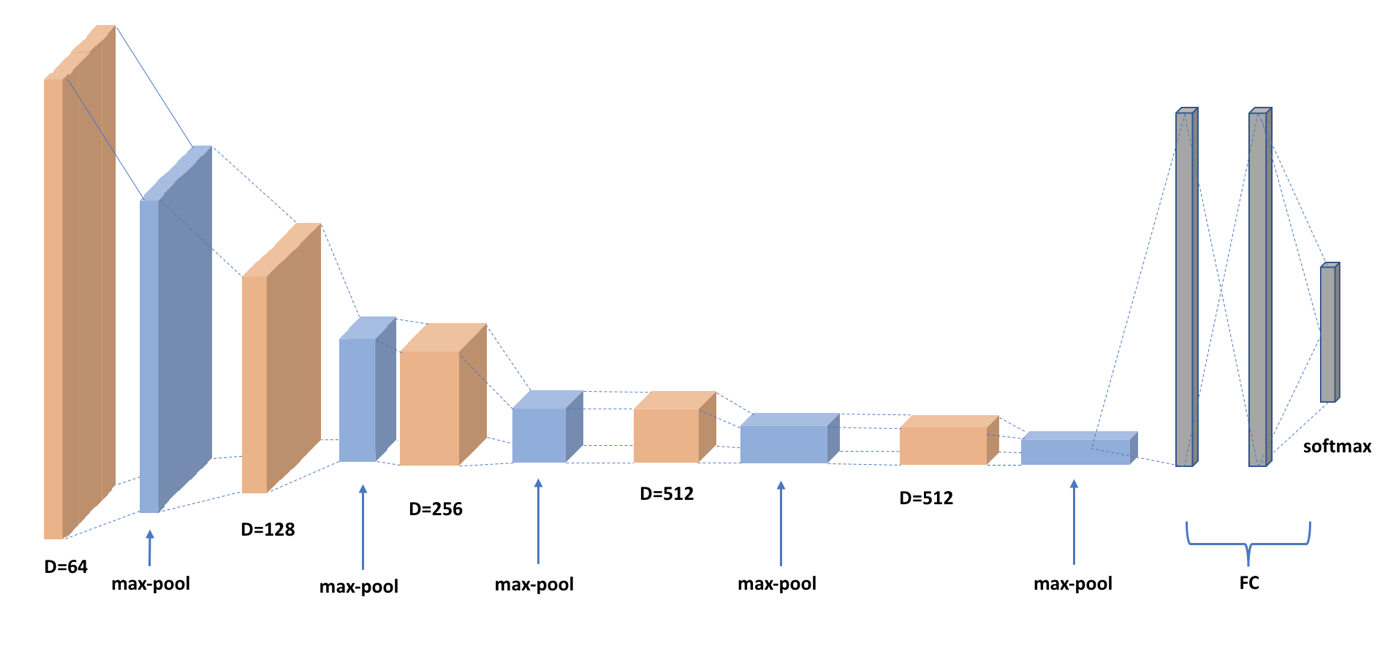


**Supplemental Figure S3. VGG convolutional neural networks.** The VGG architecture is widely used because of its simplicity. It is composed only of convolutional layers using a kernel of dimensions (W,H)= (3,3) and a stride S=1, and max-pooling layers with dimensions n x p = 2 x 2 with a stride S=2. It therefore belongs to the wider class of most used CNN. The rationale for this choice is that applying three consecutive convolutions to a fraction of image using a kernel of dimensions (3,3) is tantamount to studying the same part of the image using a convolutional kernel of dimensions (7,7). This avoids to use large convolution kernels to detect large shapes within the distribution of pixels. In addition, the successive use of three small convolutions allow to apply a greater number of activation functions and detect nonlinearities. A VGG-10 architecture was trained and used for this study, which instantiates 10 convolutional layers. This choice was made after preliminary testing, since it empirically optimizes a trade-off between the relative complexity of the detected objects (cells with kernels of a certain shape, in a strongly heterogeneous environment) and the time requirements for the study. The training requires typically less than two weeks on a four-GPU computer.

D= depth, 3x3 = convolutional kernel dimensions.The last two layers are « fully-connected » (FC) layers, where each neuron is connected to all neurons of the previous layer. These FC layers aggregate all pieces of information processed separately by browsing kernels, and provide the final result (probability vector) using a softmax activation function.


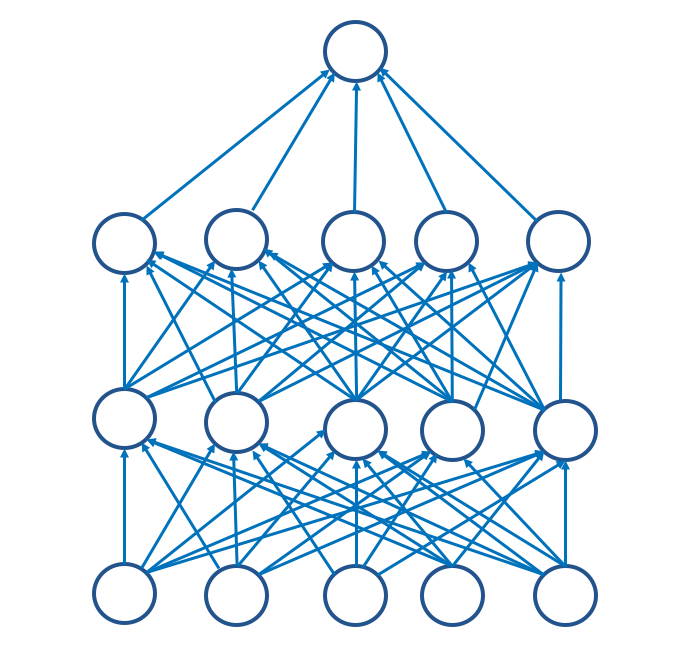

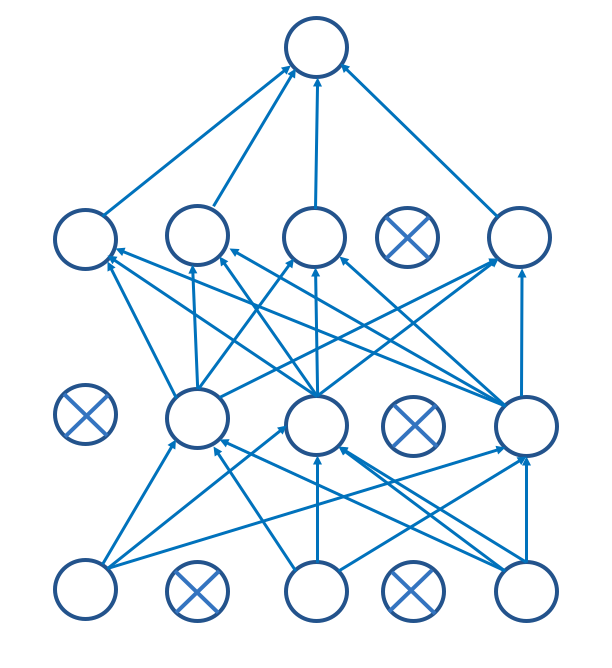


a b

**Supplemental Figure S4. Avoiding overfitting using dropout.** The effect of dropping out neurons (**b**) on a standard neural network architecture (**a**). The computational cost of training a convolutional neural network limits the practical relevance of classical machine learning techniques for preventing overfitting, as cross-validation, especially when considering several models. The so-called dropout method is considered as a powerful alternative. The principle of this regularization method is to turn off (“dropping out”) random neurons during network training. This method avoids the "co-adaptation" of neurons, namely the fact that a neuron can correct bad learning of a neuron from a previous layer. The dropout procedure is uniquely conducted during the training phase, and it is parameterized by the ratio R of randomly dropped neurons during the training, per convolutional layer. Empirical tests led us to make the usual choice R = 0.5.
